# Development of a stacking model for personalized treatment of Crohn’s disease: leveraging routine clinical features to forecast infliximab response

**DOI:** 10.3389/fphar.2026.1836369

**Published:** 2026-06-10

**Authors:** Shaojun Jiang, Limin Lin, Dan Yang, Shoutian Zhang, Desheng Zhang, Yuewen Chen, Rongfang Lin, Jianwen Xu

**Affiliations:** 1 Department of Pharmacy, The First Affiliated Hospital of Fujian Medical University, Fuzhou, China; 2 Department of Pharmacy, National Regional Medical Center, Binhai Campus of the First Affiliated Hospital, Fujian Medical University, Fuzhou, China; 3 Department of Pharmacy, Affiliated People’s Hospital of Fujian University of Traditional Chinese Medicine, Fuzhou, China

**Keywords:** crohn’s disease, infliximab, machine learning, prediction, stacking model

## Abstract

**Aims:**

Infliximab (IFX) is widely used for treating Crohn’s disease (CD), but a significant proportion of patients experience primary non-response or loss of response. Early prediction of IFX efficacy is crucial to avoid ineffective treatment, adverse effects, and financial burden. The aim of this study was to develop a stacking model using routine clinical data to predict IFX clinical response.

**Method:**

This retrospective cohort study enrolled CD patients initiating IFX therapy between January 2019 and December 2025. Feature selection was performed using statistical analysis and Least Absolute Shrinkage and Selection Operator (LASSO) regression. Base models (Elastic Net, Support Vector Machine, Random Forest, XGBoost) were built and integrated into a stacking model. Model performance was evaluated using the Area Under the Receiver Operating Characteristic Curve (AUROC), accuracy, precision, sensitivity, specificity, recall, and F-score on a hold-out testing set. Class imbalance was addressed using the Synthetic Minority Over-sampling Technique.

**Result:**

A total of 319 patients were enrolled, comprising 237 responders and 82 non-responders, reflecting a class imbalance. An independent dataset containing 43 patients was used for temporal validation. LASSO regression identified five key predictors: erythrocyte sedimentation rate, C-reactive protein, Crohn’s disease activity index, red blood cell count, and diagnostic age. The stacking model, composed of Elastic Net and Random Forest, achieved an AUROC of 0.897 (95% CI: 0.832–0.956) on the validation set and 0.874 (95% CI: 0.749–0.957) on the testing set, demonstrating robust predictive performance.

**Conclusion:**

The developed stacking model effectively predicts IFX response using readily available clinical variables, representing a preliminary step toward personalized treatment planning. Prospective validation is required before clinical implementation.

## Introduction

Crohn’s disease (CD) is a progressive immune-mediated chronic inflammatory disease. Pathogenesis may result from the interplay of environmental factors, the immune system, genetic susceptibility, and changes in the host’s microbiome, which disrupt the intestinal mucosa (Corridoni et al., 2014). The prevalence of CD has been steadily increasing in recent decades, posing significant health challenges globally, particularly with a troubling trend toward earlier onset in younger individuals (Sasson et al., 2021). Patients with CD often suffer from a range of adverse effects associated with available therapeutic agents, which severely impairs their quality of life and imposes a heavy burden on both individuals and families (Nunez et al., 2023). Therefore, developing robust and personalized therapeutic strategies is urgently needed to effectively manage this chronic ailment.

Among the available therapeutic options for CD, biologic agents such as infliximab (IFX), a tumor necrosis factor-α (TNF-α) inhibitor, have become the first-line treatment for moderate-to-severe CD due to their relatively lower risk of systemic side effects compared with traditional immunosuppressants (D'Haens and Daperno, 2006; Hemperly and Vande Casteele, 2018). Although its efficacy has been proven in several studies, many patients still experience therapeutic failure. Recent studies show that approximately 13%–40% of inflammatory bowel disease patients do not response to initial IFX therapy. During the maintenance phase, around 30%–40% of patients either fail to respond or lose their response to IFX (Billiet et al., 2014; Gibson et al., 2020). Such non-responders not only suffer from delayed treatment, potential adverse reactions, and unnecessary financial burden, but also represent a substantial waste of medical resources for healthcare systems. Therefore, early and accurate prediction of IFX efficacy in CD patients is of great clinical significance for optimizing treatment strategies and improving patient outcomes.

The pharmacokinetics of IFX in patients with CD is, however, highly variable, and a one-size-fits-all approach to dosing does not lead to similar exposures across patients, therefore, dose optimization can be challenging (Brandse et al., 2017; Kizaki et al., 2018). The mechanisms for this loss of response may be multifaceted, including genetic differences, the formation of anti-drug antibodies, variations in drug metabolism, and polymorphisms in the TNF-α gene (Matsuoka et al., 2020; Sazonovs et al., 2020). Additionally, suboptimal drug levels have also been identified as a contributing factor to therapeutic failure (Sands et al., 2022). There is increasing evidence in adults and children that adequate IFX exposure is critical when treating CD, and trough concentrations <3 μg/mL are associated with treatment failure and worse outcomes (Vermeire et al., 2024).

Predicting the response to IFX has become a key area of research in managing CD because it significantly impacts treatment outcomes and personalized medicine (Yueying et al., 2023). Identifying patients who are likely to benefit from IFX therapy in advance can greatly enhance clinical efficacy, minimize unnecessary adverse effects, and reduce healthcare costs. Although extensive research has been conducted on how IFX works in CD and the factors affecting treatment responses, there is still considerable debate about the specific reasons for variability in treatment outcomes. Current investigations have identified several critical variables that may influence the pharmacokinetics of IFX, such as body weight, anti-IFX antibodies, serum albumin levels, and C-reactive protein (CRP) concentration (Gil Candel et al., 2020; Hemperly and Vande Casteele, 2018; Yarur et al., 2023). However, the relationships between these variables are complex, involving both linear and nonlinear interactions, which makes it difficult to identify their effects on treatment response. This uncertainty underscores the urgent need for more comprehensive research to identify the factors influencing treatment responses in CD, establishing a solid evidence-based foundation for individualized treatment.

With the rapid advancement of statistical theory and computer technology, the application of machine learning in predicting treatment efficacy has emerged as a promising avenue in biomedical research (Greener et al., 2022; Hou et al., 2020). Existing studies have developed predictive methods for IFX efficacy based on specialized data sources, such as gut microbiota, metabolomics, genomics, and imaging techniques (Chen et al., 2025; Zhou et al., 2018). However, the above methods are generally limited by complex operation, long time consumption, and high cost, making them difficult to implement in routine clinical practice. Recent studies have attempted to construct IFX response predictive models using routine biomarkers: for example, a study by (Medina-Medina et al., 2023) developed a proteomics approach to identify vinculin as a potential biomarker to predict therapeutic response to biologic agents in CD, but no independent external validation cohort was used to verify the model or biomarkers. Another study used a XGBoost model with routine clinical variables, but it failed to integrate the advantages of multiple models to further improve prediction accuracy (Qiu et al., 2025).

As an advanced ensemble learning approach, the stacking model (also known as stacked generalization) can effectively integrate the prediction results of multiple basic machine learning models (e.g., random forest, support vector machine, logistic regression) and leverage the unique advantages of each model, thereby overcoming the limitations of single models and achieving superior predictive performance and robustness (Rahman et al., 2024; Zhang et al., 2024). Although machine learning-based models for predicting IFX efficacy have been reported in previous studies (Qiu et al., 2025), the application of the stacking ensemble model has not yet been explored. This study aims to identify the factors associated with therapeutic response to IFX, and to develop an interpretable machine learning model for predicting clinical response to IFX in CD patients using routine clinical examination indicators as the data source. It is expected to optimize therapeutic efficacy, reduce unnecessary adverse reactions, facilitate timely adjustment of treatment regimens, and ultimately provide evidence for early treatment decision-making and individualized therapy.

## Methods

### Study design

**FIGURE 1:**
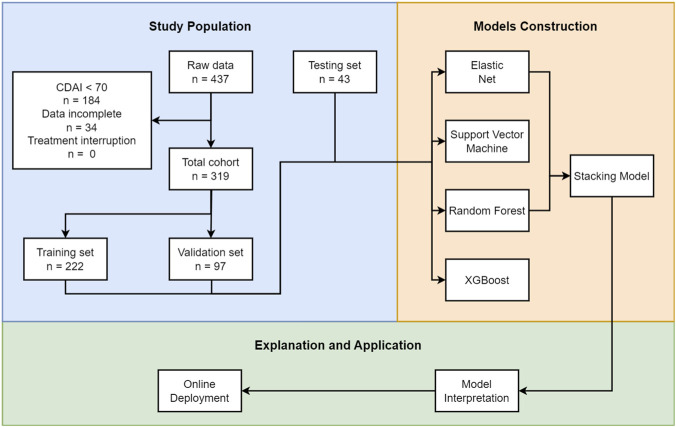
Flowchart of patient enrollment and model development.

This study utilized retrospective collected clinical data to develop multiple base models predicting clinical response to IFX in CD patients, which were then integrated into a stacking ensemble model. Subsequently, we evaluated the predictive performance of the stacking model and explored its interpretability. In this study, clinical response was defined as a decrease in CDAI score exceeding 70 within 6 months after receiving the first infliximab treatment (Thia et al., 2008). Based on this criterion, all patients were categorized into responder and non-responder groups. Patient enrollment and the study workflow are illustrated in Figure 1.

For reasons of non-interference, the Ethics Committee of the First Affiliated Hospital of Fujian Medical University granted an exemption for this study. We reported this study in accordance with the TRIPOD + AI (Transparent Reporting of a Multivariable Prediction Model for Individual Prognosis or Diagnosis, Extended for Artificial Intelligence) guidelines.

### Study population

Patients were retrospectively enrolled from the Gastroenterology Department of the First Affiliated Hospital of Fujian Medical University between January 2019 and December 2025. Patients enrolled between January 2025 and December 2025 were included as the independent testing set for temporal validation. Patient inclusion and exclusion criteria are as follows:

Inclusion criteria: (1) a diagnosis of CD established according to the consensus statement of the European Crohn’s and Colitis Organization (Maaser et al., 2019); (2) patients with CD treated by ≥ 4 scheduled IFX infusions (0, 2, 6, and followed by maintenance therapy at the same dose every 8 weeks); (3) follow-up time of at least 12 months.

Exclusion criteria: (1) crohn’s Disease Activity Index (CDAI) score below 70 prior to IFX treatment; (2) patients treated by episodic IFX therapy or who did not receive induction therapy; (3) missing more than 30% of case data or critical variables; (4) patients who were coexisting hematologic, metabolic diseases, or auto immune liver disorders; and (5) discontinuation of IFX treatment during the study or loss of follow-up.

### Data collection

The clinical information included in this study comprised demographic data (age, gender, height, weight), laboratory parameters (CRP, ESR, WBC, RBC, eGFR, albumin, ALT, AST, D-dimer, APTT, TT, PT, fibrinogen, fecal calprotectin), medications (IFX dosage, azathioprine, mesalamine, thalidomide, methotrexate), and disease characteristics (CDAI, antidrug antibodies (ADA), age at diagnosis, location, behavior, perianal disease modifiers). All predictor clinical variables were strictly collected before the first administration of infliximab. The baseline time window has also been clearly defined in Supplementary Table S1. IFX plasma concentrations were excluded as the study aimed to predict clinical response prior to administration. Samples were multiply imputed or removed based on the proportion of missing data. Detailed descriptions of missing data handling are provided in the Supplementary Appendix (Supplementary Table S2).

### Feature selection

All data were divided into training and testing sets via stratified random sampling at a 7:3 ratio. In training set, the least absolute shrinkage and selection operator (LASSO) are used for feature selection. The normality of continuous variables is verified using the Kolmogorov-Smirnov test. Normal variables are represented by mean plus or minus standard deviation, and their differences are compared using an independent sample t-test. Non-normal variables are expressed as medians (quartiles), and their differences are compared using the Mann-Whitney U test. Categorical variables are expressed as sample sizes (percentages), and their differences are compared using chi-square analysis or Fisher’s exact probability method. In LASSO regression, the Area Under Receiver Operating Characteristic Curve (AUROC) is taken as the evaluation index, and the best Lambda parameter is determined through 10-fold cross-validation. In accordance with Occam’s Razor principle, we select the maximum Lambda value within one standard deviation of the minimum error as the optimal Lambda value. Under this Lambda parameter, variables whose coefficients do not compress to zero will be included in model construction.

### Model construction and evaluation

During the data preprocessing stage, continuous variables underwent normalization transformation while categorical variables were converted to one-hot encoding. Subsequently, the synthetic minority over-sampling technique (SMOTE) based on the k-nearest neighbors was applied to address class imbalance, achieving a 1:1 sample ratio between the response and non-response groups in the training dataset.

Fundamental models were constructed using Elastic Net (EN), Support Vector Machine (SVM), Random Forest (RF), and eXtreme Gradient Boosting (XGBoost) algorithms. In this study, the positive class was defined as patients who did not respond to IFX (non-responders). Model performance was evaluated primarily using the Area Under the Receiver Operating Characteristic Curve (AUROC), along with accuracy, precision, recall, specificity, negative predictive value (NPV), and F-score. Confidence intervals for the AUROC were obtained through 1,000 bootstrap iterations. Optimal hyperparameters for each model were determined through 10-fold cross-validation and Bayesian optimization. The optimal hyperparameter combinations were internally validated on the validation set to assess their generalization performance. Subsequently, a stacking model was constructed based on the principles of the underlying models and their performance on the validation and testing sets. Finally, the stacking model was interpreted using the DALEX package. All statistical analyses and model construction were performed using R software version 4.4.1 (R Foundation for Statistical Computing, Vienna, Austria).

## Results

### Characteristics of patients

This study initially enrolled 437 patients, with 319 ultimately included after exclusion criteria application, comprising 237 responders and 82 non-responders (Figure 1). Table 1 presents baseline characteristics of responders versus non-responders. Compared with responders, non-responders had a younger median age (25.0 vs. 27.0 years, p = 0.007). Laboratory results revealed statistically significant differences between groups in CRP, ESR, WBC, RBC, albumin, ALT, AST, D-dimer, and fibrinogen (P < 0.05). The proportion of IFX doses administered was similar across groups (P = 0.345). Compared to the non-responder group, more patients in the responder group received combination therapy with thalidomide (1.2% vs. 8.4%, P = 0.020). Additionally, the median CDAI score was significantly higher in the responder group than in the non-responder group (171.4 vs. 89.5, p < 0.001). Regarding Montreal classification, differences between groups primarily concerned age at diagnosis: the non-response group had a lower proportion diagnosed before age 16 (11.0% vs. 27.8%) and a higher proportion diagnosed after age 40 (12.2% vs. 0.8%, p < 0.001). No statistically significant differences were observed in other demographic information, disease characteristics, laboratory results, concomitant medications, or Montreal features. Comparisons between the training and testing sets are shown in Supplementary Table S3 in the Supplementary Material.

**TABLE 1:** Demographic and clinical characteristics in non-responding and responding groups.

Characteristics	Non-response group N = 82	Response group N = 237	P value
Age (year)	27.0 (22.0, 33.0)	25.0 (19.0, 30.0)	0.007
Gender, n (%)	​	​	0.393
Male	49 (59.8%)	156 (65.8%)	​
Female	33 (40.2%)	81 (34.2%)	​
Height (cm)	168.0 (160.0, 173.0)	170.0 (161.0, 176.0)	0.083
Weight (kg)	53.0 (47.5, 63.0)	58.0 (48.0, 64.0)	0.074
CRP (mg/L)	5.5 (5.0, 23.2)	5.0 (5.0, 8.0)	<0.001
ESR (mm/h)	27.4 (13.0, 47.8)	16.0 (7.0, 30.0)	<0.001
WBC (×10^9^/L)	5.4 (4.3, 6.8)	6.0 (4.9, 7.2)	0.026
RBC (×10^12^/L)	4.6 (4.1, 5.0)	4.8 (4.5, 5.1)	0.001
eGFR (ml/min/1.73 m^2^)	125.3 (115.9, 135.8)	124.8 (116.6, 132.6)	0.629
Albumin (g/L)	43.1 (39.7, 45.4)	44.2 (41.9, 46.4)	0.006
ALT (U/L)	13.8 (10.0, 21.2)	16.0 (12.0, 22.0)	0.024
AST (U/L)	17.9 (15.0, 21.0)	19.0 (16.4, 23.0)	0.031
D-dimer (μg/L)	0.2 (0.1, 0.4)	0.2 (0.1, 0.3)	0.003
APTT (s)	30.5 (28.3, 33.5)	29.8 (27.1, 33.4)	0.489
TT (s)	15.7 (15.0, 16.3)	15.9 (15.2, 16.6)	0.066
PT (s)	12.2 (11.5, 12.6)	12.0 (11.5, 12.7)	0.412
Fibrinogen (g/L)	3.2 (2.5, 4.2)	2.7 (2.2, 3.2)	0.001
Fecal calprotectin (μg/g)	​	​	0.415
<15	12 (14.6%)	32 (13.5%)	​
15–60	10 (12.2%)	44 (18.6%)	​
≥60	60 (73.2%)	161 (67.9%)	​
IFX dose (mg)	​	​	0.345
200	11 (13.4%)	28 (11.8%)	​
300	47 (57.3%)	142 (59.9%)	​
400	24 (29.3%)	56 (23.6%)	​
500	0 (0.0%)	9 (3.8%)	​
600	0 (0.0%)	2 (0.8%)	​
Concomitant therapy, n (%)			
Azathioprine	36 (43.9%)	75 (31.6%)	0.061
Mesalamine	12 (14.6%)	25 (10.5%)	0.426
Thalidomide	1 (1.2%)	20 (8.4%)	0.020
Methotrexate	0 (0.0%)	9 (3.8%)	0.118
CDAI, n (%)	89.5 (78.5, 122.0)	171.4 (131.7, 225.0)	<0.001
ADA, n (%)	21 (25.6%)	45 (19.0%)	0.203
Age at diagnosis (year), n (%)	​	​	<0.001
<16	9 (11.0%)	66 (27.8%)	​
16–40	63 (76.8%)	169 (71.3%)	​
≥40	10 (12.2%)	2 (0.8%)	​
Location, n (%)	​	​	0.878
L1, ileal	1 (1.2%)	10 (4.2%)	​
L2, colonic	3 (3.7%)	9 (3.8%)	​
L3, ileocolonic	44 (53.7%)	114 (48.1%)	​
L4, isolated upper disease	2 (2.4%)	7 (3.0%)	​
L2 + L4	1 (1.2%)	3 (1.3%)	​
L3 + L4	31 (37.8%)	94 (39.7%)	​
Behavior, n (%)	​	​	0.494
Non-stricturing, non-penetrating	38 (46.3%)	132 (55.7%)	​
Stricturing	35 (42.7%)	83 (35.0%)	​
Penetrating	6 (7.3%)	15 (6.3%)	​
Stricturing and penetrating	3 (3.7%)	7 (3.0%)	​
Perianal disease modifiers, n (%)	52 (63.4%)	156 (65.8%)	0.795

CRP: C-reactive Protein; ESR: erythrocyte sedimentation rate; WBC: white blood cell; RBC: red blood cell; eGFR: estimated glomerular filtration rate; ALT: alanine aminotransferase; AST: aspartate aminotransferase; APTT: activated partial thromboplastin time; TT: thrombin time; PT: prothrombin time; IFX, infliximab; CDAI: Crohn’s Disease Activity Index; ADA, antidrug antibodies.

### LASSO regression

**FIGURE 2:**
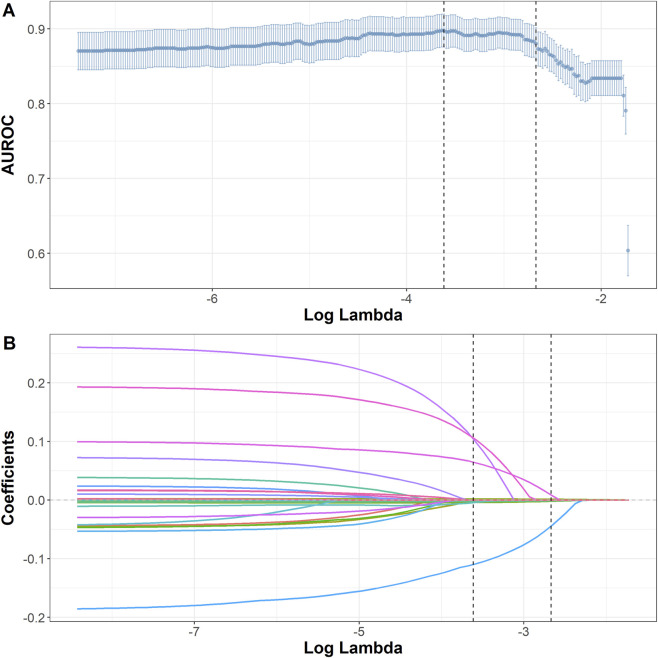
Selection of variables in LASSO regression. **(A)** Cross-validation curve for tuning the penalty parameter λ. The left vertical dashed line represents λ.min (the value with minimum binomial deviance), and the right line represents λ.1se. λ.1se = 0.0692 was selected for model sparsity. **(B)** Coefficient trace plot showing how each variable’s coefficient shrinks toward zero as λ increases. Five variables with non-zero coefficients at the selected λ were retained: ESR, CRP, CDAI, RBC, and age at diagnosis. CDAI: Crohn’s Disease Activity Index; CRP: C-Reactive Protein; ESR: Erythrocyte Sedimentation Rate; RBC: Red Blood Cell.

Figure 2 displays the results of the LASSO regression. With Lambda = 0.0692 as the optimal parameter, the coefficients for ESR, CRP, CDAI, RBC, and age at diagnosis were −0.00133, −0.00208, 0.00138, 0.00879, and −0.04433, respectively. Consequently, these five variables were incorporated into model construction.

### Performance of models

Four models—EN, SVM, RF and XGBoost—were constructed using five indicators as dependent variables: ESR, CRP, CDAI, RBC, and diagnostic age. The AUROC values for internal and temporal validation across all four models are shown in Table 2. All models performed well on the validation set (AUROC >0.85). However, in testing set, EN, RF, and SVM showed slight overfitting, with a decrease in AUROC. XGBoost showed the most stable prediction results, with the smallest gap between validation set and testing sets (AUROC = 0.875 and 0.876).

**TABLE 2:** Performance of models in validation set and testing set.

Model	Cohort	Accuracy	Precision	Recall	Specificity	NPV	F Score	AUROC (95% CI)
EN	Validation	0.835	0.629	0.880	0.819	0.952	0.733	0.899 (0.827–0.958)
EN	Test	0.767	0.556	0.833	0.742	0.920	0.667	0.844 (0.723–0.946)
SVM	Validation	0.804	0.583	0.840	0.792	0.934	0.689	0.889 (0.810–0.952)
SVM	Test	0.767	0.556	0.833	0.742	0.920	0.667	0.860 (0.737–0.953)
RF	Validation	0.856	0.704	0.760	0.889	0.914	0.731	0.883 (0.802–0.949)
RF	Test	0.767	0.556	0.833	0.742	0.920	0.667	0.868 (0.758–0.965)
XGBoost	Validation	0.825	0.654	0.680	0.875	0.887	0.667	0.875 (0.800–0.939)
XGBoost	Test	0.721	0.500	0.750	0.710	0.880	0.600	0.876 (0.759–0.962)

EN: elastic net regression; RF: random forest; SVM: support vector machine; XGBoost: eXtreme Gradient Boosting; NPV: negative predictive value; AUROC: area under receiver operating characteristic curve; CI: confidence interval.

The remaining performance metrics for all models on the testing set are summarized in Table 2. After comprehensively evaluating both the underlying principles and performance metrics of the models, we constructed a stacking model whereby EN and RF served as the base-learner.

### Performance of stacking model

The performance of the stacking model on the validation set and testing set is shown in Table 3. The stacking model achieved an AUROC of 0.897 on the validation set, outperforming all base models except EN. Meanwhile, its performance on the testing set was second only to XGBoost (AUROC 0.897 vs. 0.899). Additionally, it is worth noting that the specificity of the stacking model was greater than 0.90 in both the training and testing sets.

**TABLE 3:** Performance of stacking model in validation set and testing set.

Model	Cohort	Accuracy	Precision	Recall	Specificity	NPV	F Score	AUROC (95% CI)
Stacking	Validation	0.835	0.737	0.560	0.931	0.859	0.636	0.897 (0.832–0.956)
Stacking	Test	0.767	0.571	0.667	0.806	0.862	0.615	0.874 (0.749–0.957)

NPV: negative predictive value; AUROC: area under receiver operating characteristic curve; CI: confidence interval.

**FIGURE 3:**
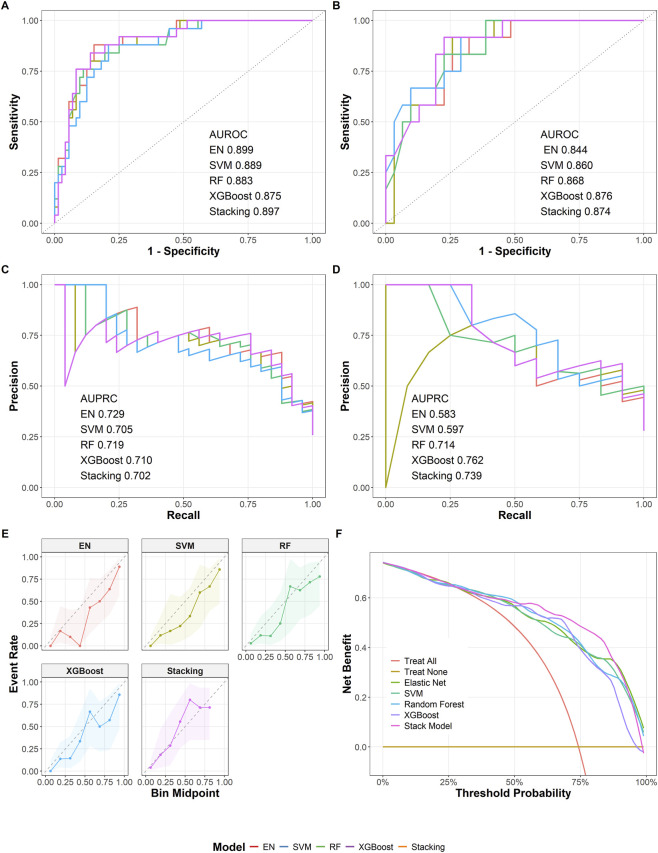
Performance comparison of five models (EN, SVM, RF, XGBoost, and Stacking) for predicting IFX clinical response. **(A,B)** AUROC with 95% confidence intervals (bootstrap = 1,000) on the validation set **(A)** and testing set **(B)**. **(C,D)** AUPRC on the validation set **(C)** and testing set **(D)**. **(E)** Calibration curves showing the agreement between predicted probabilities and observed frequencies. Perfect calibration follows the diagonal dashed line. **(F)** Clinical decision curves plotting net benefit against threshold probability. The stacking model (pink line) shows superior net benefit in the 55%–85% threshold range. EN: Elastic Net; RF: Random Forest; SVM: Support Vector Machine; XGBoost: Extreme Gradient Boosting.

Subsequently, we compared the stacking model with the baseline model. Figures 3A,B show no significant differences in the curve characteristics among the models. Delong’s test also indicates that the stacking model demonstrates non-inferiority compared to the baseline model (P > 0.05). Figures 3C,D show that the AUPRC values for EN and SVM vary significantly between the validation and test sets. In contrast, the performance of RF, XGBoost, and the stacking model was relatively stable. Figure 3E presents the calibration curves for each model. EN exhibited a noticeable underestimation in the range of 0.25–0.5. The other models generally did not show any significant bias. As seen in the clinical calibration curves, when the prediction threshold ranged from 55% to 85%, the stacking model demonstrated a higher net benefit compared to the other models (Figure 3F).

Considering the objective of this study—to identify the population unresponsive to IFX—and the comparative results of the model metrics (particularly specificity and the clinical decision curve), we selected the stacking model as the final model.

### Exploratory subgroup analysis

Due to sample size considerations, we conducted a subgroup analysis of the stacking model’s performance on the validation set to explore model fairness. Results are shown in Table 4. When CDAI <150, the model’s predictive accuracy decreased (AUROC = 0.754). Due to the limited sample size in certain subgroups (e.g., age ≥40 years, n = 4), no definitive conclusions regarding subgroup fairness can be drawn. The following results should be interpreted as hypothesis-generating and require validation in larger, more balanced cohorts. It should be noted, however, that due to the lack of patients with CDAI >450, further evaluation of the model’s performance in this patient group is warranted (Collins et al., 2024).

**TABLE 4:** Exploratory analysis of stacking model in validation set.

Subgroup	Total	Non-response	Accuracy	Precision	Recall	Specificity	NPV	F Score	AUROC
Age									
<16	22	2	0.909	-	0.000	1.000	0.909	-	1.000
16–40	71	19	0.803	0.667	0.526	0.904	0.839	0.588	0.854
≥40	4	4	1.000	1.000	1.000	-	-	1.000	-
Gender									
Male	64	19	0.828	0.833	0.526	0.956	0.827	0.645	0.897
Female	33	6	0.848	0.571	0.667	0.889	0.923	0.615	0.914
CDAI									
<150	46	21	0.739	0.765	0.619	0.840	0.724	0.684	0.754
150–220	30	3	0.900	0.500	0.333	0.963	0.929	0.400	0.975
221–450	21	1	0.952	-	0.000	1.000	0.952	-	1.000
>450	0	0	-	-	-	-	-	-	-

NPV: negative predictive value; AUROC: area under receiver operating characteristic curve; CI: confidence interval.

### Model interpretation

**FIGURE 4:**
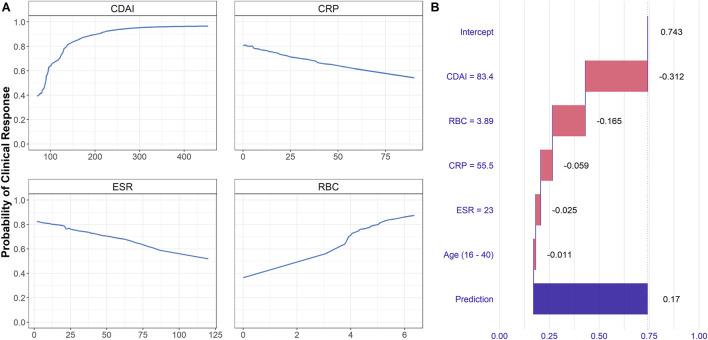
Global and local interpretability of the stacking model using break-down plots. **(A)** Global variable importance: average absolute contribution of each feature to the model’s prediction across all patients. **(B)** Local explanation for a single patient. The horizontal bars represent the sequential contribution of each variable to the final prediction, starting from the intercept. The final prediction (predicted probability of being a responder) is marked by the vertical dashed line. CDAI: Crohn’s Disease Activity Index; CRP: C-Reactive Protein; ESR: Erythrocyte Sedimentation Rate; RBC: Red Blood Cell.

**FIGURE 5:**
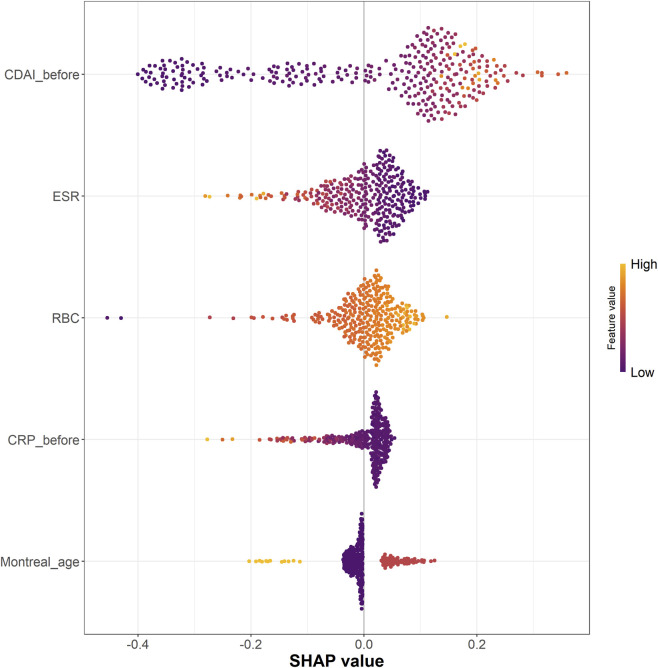
SHAP summary plot for the stacking model. Each dot in the plot corresponds to one patient in the validation set. Color encodes the feature value, with yellow indicating higher values and purple indicating lower values. The x-axis displays the SHAP value, where positive values are associated with an increased predicted probability of response. Features are arranged in descending order of importance from top to bottom.

Figure 4A presents the overall influence of model variables on outcomes. Specifically, CDAI correlates positively with clinical response rates within the 100–250 range, after which it enters a plateau phase. CRP, ESR, and RBC exhibit relatively linear effects on model outcomes, with ESR showing a negative correlation. Clinical response rate decreased with increasing Montreal Age and showed significant stratification across subgroups (Supplementary Figure S2). Figure 4B illustrates the model prediction process at the individual level. Additionally, the plot of SHAP-based feature importance similarly reflects the above results (Figure 5).

## Discussion

The response to IFX, a monoclonal antibody used in treating CD, is influenced by multiple clinical variables. This study developed an IFX efficacy prediction model using stacking model techniques based on retrospective data from a single center. The model achieved ideal predictive capability while incorporating only five variables. Additionally, we systematically explained the prediction process of the model from both global and individual levels, which may help clinicians understand model predictions, but clinical utility remains to be proven. In terms of model performance evaluation, the stacking model achieved high specificity (validation: 0.931; test: 0.806) and high NPV (0.931 and 0.806, respectively), indicating that it reliably identifies true responders. In contrast, the moderate precision (0.737 and 0.571) and recall (0.560 and 0.667) suggest that when the model predicts a non-responder, further evaluation is warranted before making treatment adjustments.

While the CDAI has been one of the most commonly used scoring systems for measuring disease activity, the prognostic value of CDAI remains controversial. As a composite metric integrating patient-reported outcomes and clinical biomarkers in CD, CDAI’s predictive capacity for biologic efficacy was previously unsubstantiated. In this study we identify CDAI as the most significant factor in the prediction model for assessing clinical response to IFX. This finding highlights the importance of monitoring disease activity scores during IFX therapy, as neglecting CDAI may result in poor clinical decision-making.

The influence of CDAI on IFX response may be attributed to its comprehensive assessment of various clinical parameters, including patient-reported symptoms and physical examination findings (Dubinsky et al., 2023). High CDAI score reflects greater inflammation and disease severity, which may not only predict poor response to therapy but also suggest that patients might require more aggressive treatment approaches or alternative therapies (Dubinsky et al., 2023; Peyrin-Biroulet et al., 2022). Similar to this study, multiple studies have also used CDAI as a predictor in their models (Peng et al., 2023). However, the potential circularity between baseline CDAI and the outcome definition (≥70-point decrease) is a concern. Therefore, we evaluated all base models after removing CDAI from the feature set (Supplementary Table S4). Excluding CDAI reduced AUROC values across all models by 0.062–0.206 on both the validation and testing sets. Nonetheless, the remaining features (ESR, CRP, RBC, and age at diagnosis) retained non-negligible predictive discrimination, with AUROC values ranging from 0.65 to 0.81 after CDAI removal. Incorporating CDAI into predictive models provides valuable insights for assessing IFX responses and guiding personalized treatment plans for inflammatory diseases.

Our study also found that baseline CRP counts and ESR levels significantly influenced the clinical response to IFX. The predictive value of CRP and ESR for IFX efficacy has been extensively and thoroughly studied (Constantine-Cooke et al., 2025; Qiu et al., 2025). This study is consistent with previous research findings. In the study by Buderus et al., in 90% of children with active CD, the CRP level was within unnormal level. Inflammatory markers may also be elevated in a variety of other disorders, especially infectious diseases (Buderus et al., 2015). Nonetheless, these markers show low sensitivity and specificity in differentiating CD from other gastrointestinal diseases or in evaluating disease activity, the clinical usefulness of their isolated testing is limited. Taken together, the assessment of clinical symptoms and combined use of blood-based non-invasive inflammatory biomarkers may be useful in evaluating the therapeutic effect of infliximab, significant mucosal inflammation may require adjustment of the dose of infliximab (Daniluk et al., 2019). Additionally, laboratory factors, such as liver function tests, did not significantly affect the effectiveness of IFX in CD patients. Unlike other small-molecule drugs, its disposition may not be predictable by markers of liver function (such as ALT, AST).

Regarding predictive factors, to our knowledge, there appears to be no published literature indicating that RBC can serve as a predictor of IFX efficacy. The only closely related evidence stems from a previous study identifying an association between hemoglobin dynamic clusters and long-term therapeutic response, which demonstrated that patients with elevated baseline hemoglobin presented with markedly superior response rates at the 52-week follow-up (Qiu et al., 2025). Hemoglobin is predominantly localized within red blood cells, and erythrocytes function as the fundamental carrier for hemoglobin storage and transportation. Traditionally well-documented as oxygen carriers, RBCs exert indispensable functions in regulating innate immunity. Mechanistically, RBCs constitutively express Toll-like receptor 9 and are capable of scavenging excessive circulating host cell-free DNA. The capture of cell-free DNA by RBCs facilitates the accelerated clearance of erythrocytes and consequently triggers sustained inflammatory activation (Lam et al., 2024). In our study we have explained how RBC levels numerically influence the model’s decision-making process. These findings emphasize that dynamic alterations in routine blood measurements may provide enhanced predictive capabilities, potentially guiding posttreatment clinical decisions.

Loss of response to anti-TNF therapy poses a significant challenge due to limited therapeutic options. Insufficient drug exposure may contribute to a loss of therapeutic response, which can result from the formation of antidrug antibodies (ADAs) (Bar-Yoseph et al., 2019; Bitoun et al., 2023). ADAs can affect the effectiveness of therapeutic proteins in several ways, including directing against the biologically active site, reducing therapeutic target recognition (anti-idiotypic or neutralizing antibodies), or bind to other parts of the molecule (non-neutralizing antibodies) (Hemperly and Vande Casteele, 2018). Both neutralizing and non-neutralizing antibodies can affect therapeutic drug exposure by enhancing clearance of the medication and reducing its circulating half-life. Notably, the ADAs against IFX are predominantly neutralizing antibodies (Krishna and Nadler, 2016).

However, our study did not confirm these findings. Not all ADAs influence therapeutic effectiveness or have clinical significance (Dotan et al., 2014). Research conducted across various cohorts has indicated that the antibody response to IFX and its effects on pharmacokinetics may be transient (Ungar et al., 2014; Vande Casteele et al., 2013). Patients with transient ADAs were less likely to discontinue IFX compared to those with sustained ADAs, who had significantly higher ADA levels and a fivefold increased risk of discontinuation due to persistent loss of response or intolerance (Vande Casteele et al., 2013). This variability is influenced by intrinsic patient factors and multiple drug-related factors that determine immunogenicity towards a therapeutic antibody (Hindryckx et al., 2017).

In this analysis, concomitant immunomodulators such as azathioprine did not significantly affect IFX pharmacokinetics. This finding is significant because concomitant immunomodulators have been shown to affect the efficacy of IFX in other therapeutic areas, such as rheumatoid arthritis (Ruffolo et al., 2010; Van Assche et al., 2008; Yarur et al., 2015). They are also known to positively correlate with IFX trough levels. However, our study did not replicate these observations. The reason for this disparity remains unclear, but our results align with another study that reported no significant impact of various medications several drugs, including immunomodulators, on IFX pharmacokinetics parameters in ankylosing spondylitis patients (Xu et al., 2008). One explanation for the differences between our findings and those of earlier investigations could be due to the limited number of patients receiving IFX monotherapy in our study. These findings underscore the necessity for further exploration into the efficacy characteristics of IFX across various diseases or therapeutic settings.

Among the variables not included in the model, the most noteworthy is FC, which has progressively become a primary or secondary outcome measure in clinical studies (Janssen et al., 2023). Some studies have also identified FC as a predictor of IFX efficacy and indicates that an FC greater than 250 μg/g is a significant predictor of loss of response to IFX (Roblin et al., 2017). However, this study did not observe any correlation between FC and clinical response to IFX. A possible reason is that the overall FC levels in the study population were not high, with the vast majority below 100 μg/g.

Medical costs remain a significant consideration in CD treatment. Direct medical expenses for CD have steadily increased over the past decade, with TNF-α inhibitors accounting for 70% of total treatment costs (Targownik et al., 2020). A European study revealed that over a 5-year follow-up period, the annual cost per patient with Crohn’s disease reached 3,542 euros (Burisch et al., 2020). This expenditure remains a heavy burden on the healthcare system. Early identification of non-responders within the population can reduce this expenditure to some extent. Simultaneously, early prediction can reduce the health losses and time costs patients endure from repeated attempts at ineffective therapies. Since the model incorporates only routine diagnostic tests and clinical evaluation criteria for CD patients—avoiding complex and costly approaches like pharmacokinetics, gut microbiology, metabolomics, and genomics—this also facilitates broader model implementation.

Several limitations should be acknowledged in this study. Firstly, a fundamental limitation is the inherent coupling between the outcome (≥70-point CDAI decrease) and baseline CDAI, which was the strongest predictor. As shown in Table 1, responders had significantly higher baseline CDAI than non-responders (171.4 vs. 89.5, p < 0.001). Thus, the model’s reliance on baseline CDAI may reflect greater room for improvement rather than true pharmacological efficacy, potentially inflating its predictive performance. Secondly, the stacking model simulation capability in this study depends on high-quality input data, including timely CDAI assessments, which presents challenges for integrating real-world data. Our study was a single-center retrospective design, the relatively homogeneous patient population may not fully represent the diverse clinical characteristics of CD patients receiving IFX therapy in other regions or healthcare settings and may limit the generalizability of the developed model. Therefore, the most prudent and a prudent approach before clinical deployment is to conduct minor calibration or validation using local data from the target hospital. Thirdly, the model only included routine clinical and laboratory variables, whereas emerging predictive factors such as pharmacogenetic data, metabolomic profiles, and gut microbiota were not integrated. This may restrict the model’s incremental value compared with some biomarker-based models. Despite these limitations, our study presents still provides a valuable reference for clinical decision-making in precision therapy for CD patients. Furthermore, large-sample, multicenter, randomized prospective studies with long-term follow-up and comprehensive outcome assessments are warranted to validate the model’s clinical utility, long-term efficacy, safety profile, and cost-effectiveness, thereby facilitating its translation into routine clinical practice and advancing individualized care for CD patients.

## Conclusion

We constructed a stacking model using five routine clinical variables to predict IFX response in Crohn’s disease. The model showed promising internal (AUROC 0.897) and exploratory temporal (AUROC 0.874) performance, with high specificity for identifying non-responders. However, due to single-center retrospective data and limited temporal validation (n = 43), this model should be considered a tool to assist clinical decision-making. Prospective multi-center studies are required before any clinical application.

## Data Availability

The R scripts used in this study and the final model file have been released at https://github.com/simpleseasalt/IFX_effecacy. The final model is deployed on http://simplydeploy.work/IFX_efficiency/. The original contributions presented in the study are included in the article/Supplementary Material, further inquiries can be directed to the corresponding author.
