# Supplementary material for: Development of a stacking model for personalized treatment of Crohn’s disease: leveraging routine clinical features to forecast infliximab response
Source: Front Pharmacol. 2026 Jun 10;17:1836369. doi: 10.3389/fphar.2026.1836369 (PMC13290758; doi:10.3389/fphar.2026.1836369)
Supplement: Supplementary file 1 [file DataSheet1.docx]

## Supplementary Materia

## Table S1: Timing of Measurements for The Variables Included in The Study.

| Variables | Measurement timing |
| --- | --- |
| Age | At enrollmentx |
| Gender | At enrollment |
| Height | At enrollment |
| Weight | At enrollment |
| CRP | 3 days before IFX therapy |
| ESR | 3 days before IFX therapy |
| WBC | 3 days before IFX therapy |
| RBC | 3 days before IFX therapy |
| eGFR | 3 days before IFX therapy |
| Albumin | 3 days before IFX therapy |
| ALT | 3 days before IFX therapy |
| AST | 3 days before IFX therapy |
| D-dimer | 3 days before IFX therapy |
| APTT | 3 days before IFX therapy |
| TT | 3 days before IFX therapy |
| PT | 3 days before IFX therapy |
| Fibrinogen | 3 days before IFX therapy |
| Fecal calprotectin | 3 days before IFX therapy |
| Infliximab dose | At enrollment |
| Concomitant therapy | The entire course of treatment |
| CDAI | 3 days before and 6 months after IFX therapy |
| ADA | At enrollment |
| Age at diagnosis | At enrollment |
| Location | At enrollment |
| Behavior | At enrollment |
| Perianal disease modifiers | At enrollment |

CDAI: Crohn's Disease Activity Index; CRP: C-reactive Protein; ESR: Erythrocyte Sedimentation Rate; WBC: White Blood Cell; RBC: Red Blood Cell; eGFR: Estimated Glomerular Filtration Rate; ALT: Alanine Aminotransferase; AST: Aspartate Aminotransferase; APTT: Activated Partial Thromboplastin Time; TT: Thrombin Time; PT: Prothrombin Time.

## Handle missing data

Missing data were handled using different methods based on their proportion, as shown in Table S2. The specific process for multiple imputation is as follows: using the completed variables as predictors and the missing variables as response variables, a random forest model was employed to predict the missing values. This created five complete datasets, each containing different plausible imputed values. Subsequently, the mean or mode of the five imputed values was adopted as the final imputed value.' In addition, we have also provided the missing proportions of all variables (Figure S1) and the corresponding handling methods (Table S2).

## Figure S1: Missing Percent of Variables.


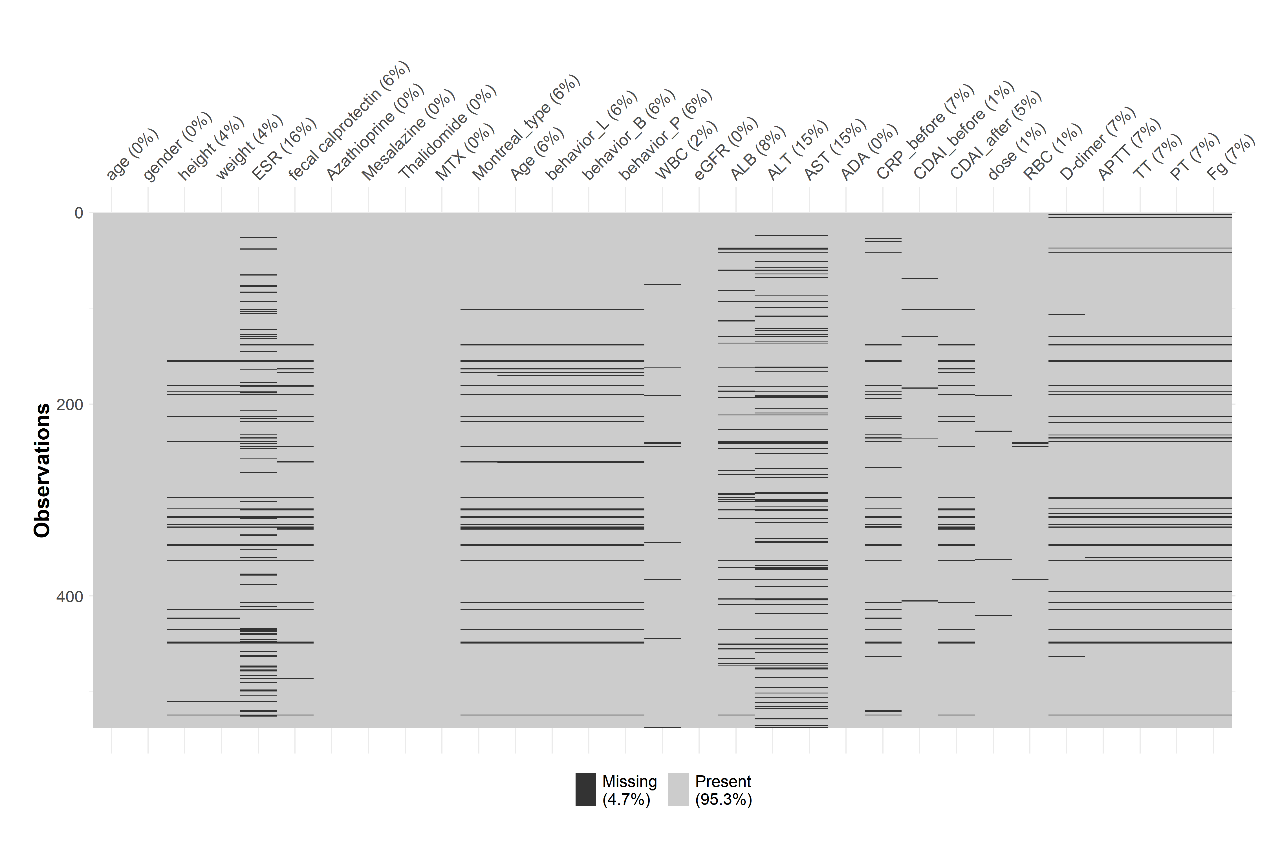


## Table S2: Methods for Handling Missing Data.

| Missing percent | Methods | Variable |
| --- | --- | --- |
| < 5% | Imputed with median or mode | CRP_before, D_dimer, APTT, TT, PT, Fg, WBC, height, weight, dose, RBC, fecal_calprotectin, Montreal_age, Montreal_L, Montreal_B, Montreal_P, monitoring_c |
| 5% - 30% | Multiple imputation | ALT, AST, ESR, ALB |
| ≥ 30% | Discard the sample | None |

## Table S3：Predictors in Training Set and Testing Set.

| Characteristics | Training set N = 222 | Testing set N = 43 | P value |
| --- | --- | --- | --- |
| CRP (mg/L) | 5.0 (5.0, 11.9) | 5.0 (5.0, 17.3) | 0.678 |
| ESR (mm/h) | 21.0 (9.0, 32.0) | 21.0 (8.0, 40.0) | 0.977 |
| RBC (×10^12/L) | 4.7 (4.4, 5.0) | 4.7 (4.1, 5.0) | 0.624 |
| CDAI | 154.1 (97.0, 206.6) | 164.2 (83.4, 225.0) | 0.898 |
| Age at diagnosis (year), n (%) |  |  | 0.003 |
| < 16 | 53 (23.9%) | 6 (14.0%) |  |
| 16 - 40 | 161 (72.5%) | 30 (69.8%) |  |
| ≥ 40 | 8 (3.6%) | 7 (16.3%) |  |

CRP: C-reactive Protein; ESR: Erythrocyte Sedimentation Rate; RBC: Red Blood Cell; eGFR: CDAI: Crohn’s Disease Activity Index.

**Table S4: Impact of baseline CDAI on AUROC of base models.**

| Model | Dataset | AUROC without CDAI | AUROC with CDAI |
| --- | --- | --- | --- |
| EN | Validation | 0.696 (0.561 - 0.821) | 0.899 (0.831 - 0.958) |
| EN | Testing | 0.804 (0.607 - 0.964) | 0.866 (0.696 - 0.982) |
| SVM | Validation | 0.694 (0.558 - 0.817) | 0.889 (0.818 - 0.952) |
| SVM | Testing | 0.813 (0.616 - 0.964) | 0.875 (0.714 - 0.982) |
| RF | Validation | 0.667 (0.535 - 0.792) | 0.883 (0.801 - 0.948) |
| RF | Testing | 0.705 (0.464 - 0.929) | 0.871 (0.723 - 0.991) |
| XGBoost | Validation | 0.652 (0.518 - 0.781) | 0.867 (0.778 - 0.937) |
| XGBoost | Testing | 0.723 (0.482 - 0.938) | 0.884 (0.732 - 0.991) |

## Figure S2: The Effect of Montreal Age on Clinical Response Rates of Infliximab


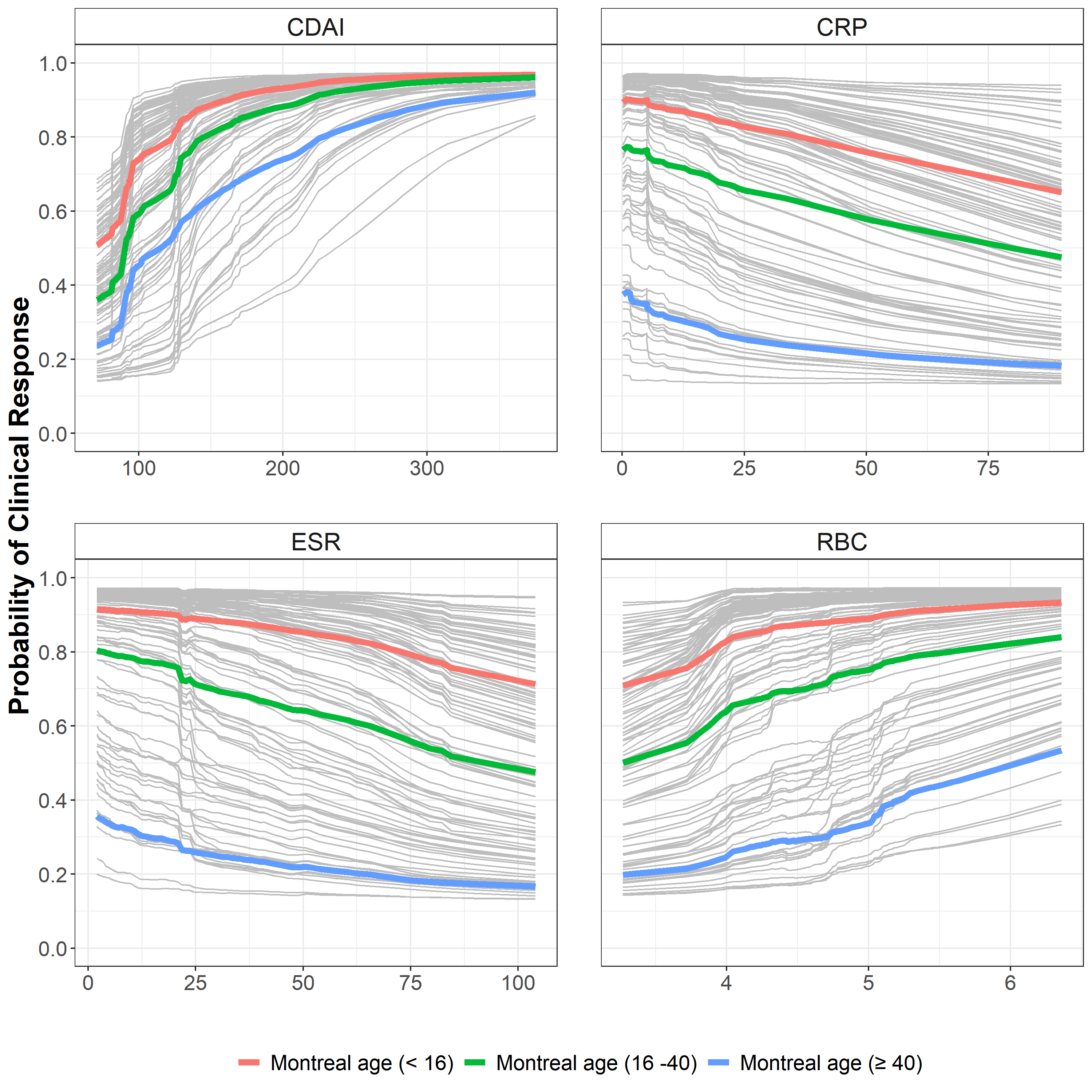


## Figure S3: Confusion Matrices of All Models on The Validation (A) and Testing (B) Sets.


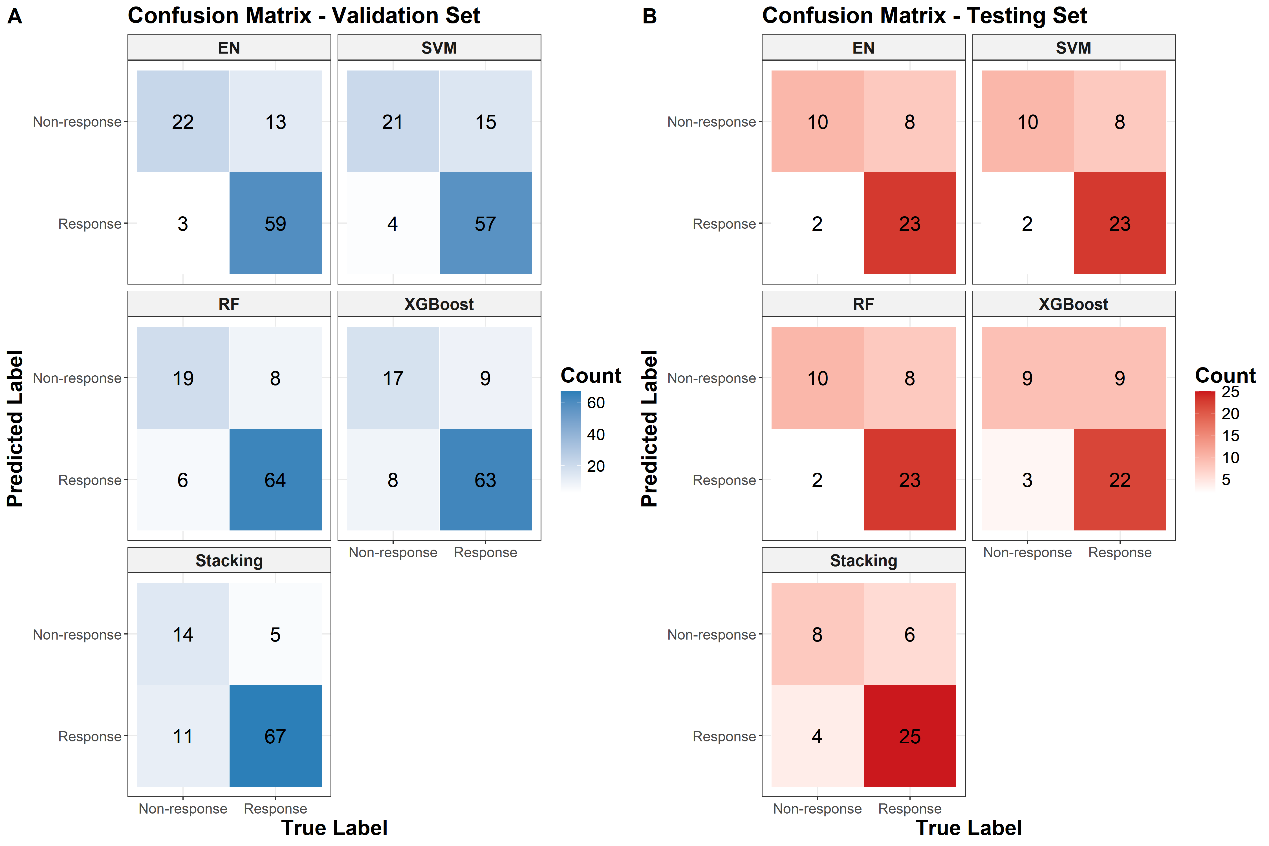


## Related Software

| Package | Version |
| --- | --- |
| DALEX | 2.4.3 |
| DALEXtra | 2.3.0 |
| glmnet | 4.1-8 |
| kernlab | 0.9-33 |
| mice | 3.16.0 |
| naniar | 1.1.0 |
| nortest | 1.0-4 |
| probably | 1.0.3 |
| randomForest | 4.7-1.2 |
| R-base | 4.4.1 |
| rstatix | 0.7.2 |
| stacks | 1.0.5 |
| themis | 1.0.2 |
| tidymodels | 1.2.0 |
| xgboost | 1.7.8.1 |
